# Noninvasive Technique for Measuring Central Venous and Arterial Pressure Using Controlled Compression Sonography

**DOI:** 10.3400/avd.oa.20-00058

**Published:** 2020-12-25

**Authors:** Hiroshi Tomoeda, Kentaro Sawada, Shingo Chihara

**Affiliations:** 1 Department of Cardiovascular Surgery, Chikugo City Hospital, Chikugo, Fukuoka, Japan; 2 Department of Surgery, Saiseikai Futsukaichi Hospital, Chikushino, Fukuoka, Japan; 3 Department of Cardiovascular Surgery, Yokokura Hospital, Miyama, Fukuoka, Japan

**Keywords:** venous pressure, central venous pressure, arterial pressure, noninvasive measurement, ultrasonography

## Abstract

**Objective:** Devices that can noninvasively measure central and peripheral venous pressures with relative ease and in a short time were developed, but the resolution of the data that can be recorded with these devices is limited to 50 mmHg.

**Materials and Methods:** We aimed to develop a system that could overcome this limitation. We used an innovative noninvasive controlled compression sonography device that could theoretically measure pressures higher than 200 mmHg. First, to validate the accuracy of our device, an in vitro study was conducted. Then, the values measured by our system were compared to conventionally obtained measurements of central venous, peripheral venous, and brachial artery pressures. Finally, regression analyses were used to determine the correlations between measurements obtained from different devices.

**Results:** With our device, the measurement of venous and arterial pressures required only 3 to 15 sec. All regression analyses revealed a significant statistical correlation between measurements, although the correlation coefficient was relatively low for arterial pressure.

**Conclusion:** For venous pressure, our system can provide measurements that could not be measured noninvasively with conventional methods. Regarding arterial pressure, although our system could measure systolic pressure, further studies are needed to confirm the clinical efficacy of our device.

## Introduction

Central venous pressure (CVP) is used to detect blood volume overload/inadequacy and can be used to determine an appropriate intensive care plan.^1,2)^ However, CVP measurements typically require aseptic catheter insertion, which is invasive, painful, time consuming, and associated with potentially serious complications, such as catheter-related bloodstream infection (CRBSI). Furthermore, CRBSI leads to high economic losses.^3)^

Several groups attempted to develop noninvasive techniques to measure CVP using ultrasonography. For example, Thalhammer et al.^1)^ developed a customized pressure manometer that is simple to use; however, the device was combined with an echo probe, and it might be difficult to produce at a low cost. Other groups reported other techniques, but one of the approaches requires a change in head position during the measurement and consists of several separate procedures.^2)^ The other method yields underestimates of the actual value.^4)^ Therefore, the existing noninvasive measurement systems exhibit limitations, and none of them can be used to measure arterial pressure. If a device can measure both arterial and venous pressure and could be created at a lower cost, it could be more widely used. The present study aimed to develop a system that could address these issues and be implemented in a variety of practices.

## Materials and Methods

After designing the prototype system, we recruited 17 healthy Japanese volunteers and 14 patients who were being treated at our intensive care unit with a central venous catheter (CVC). Next, the prototype system was evaluated using three scenarios that involved comparing the values from our system and from conventional pressure measurement methods.

The study protocol was approved by the ethics committee of Chikugo City Hospital with approval number 2016-06, and all participants provided their written informed consent to participate.

### Prototype development

**Figure 1:**
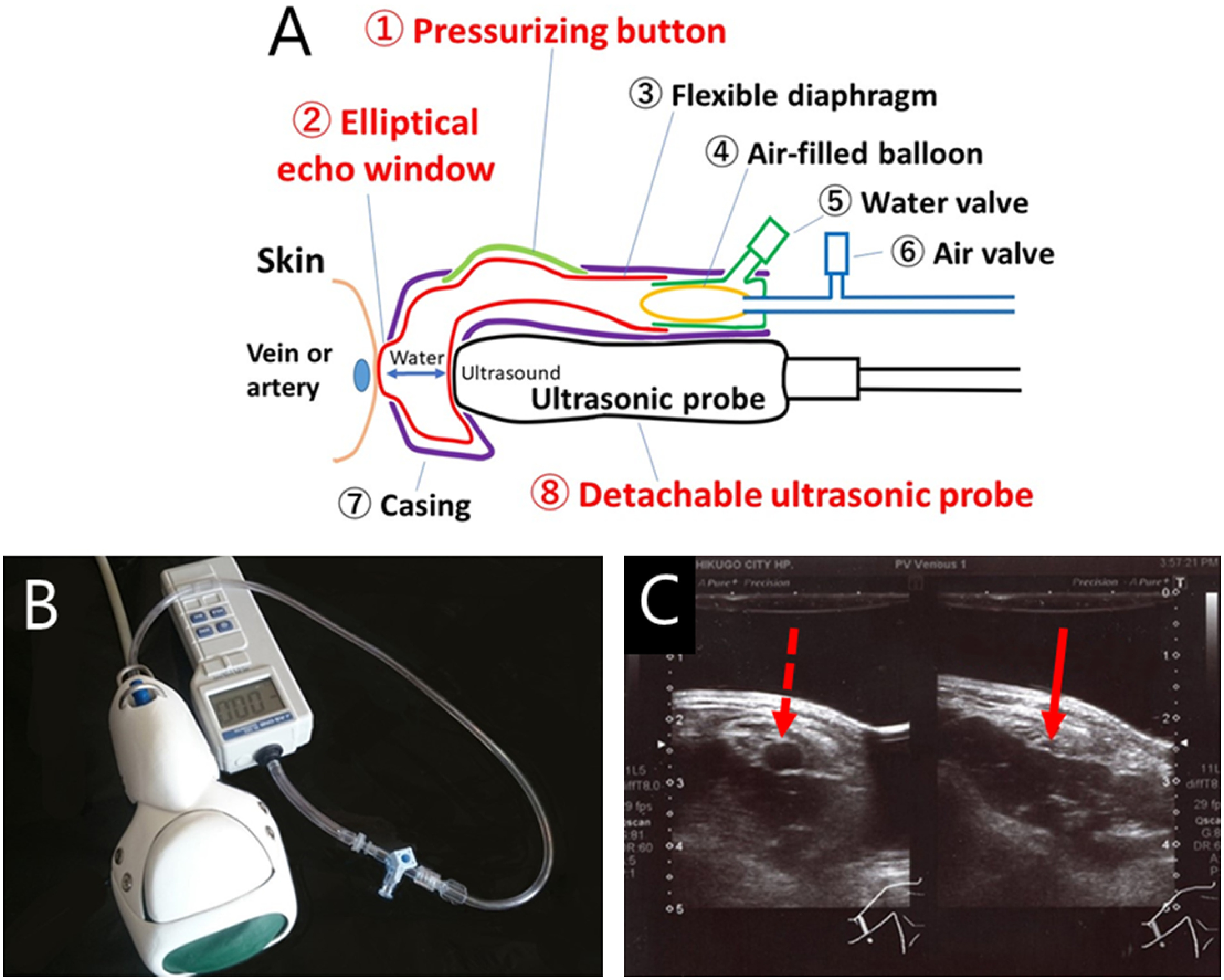
Fig. 1 (**A**) Noninvasive system for measuring venous/arterial pressure. ①Pressurizing button to increase fluid pressure to at least 200 mmHg. ②Modified elliptical window and projecting diaphragm. ③Flexible diaphragm (filled with ultrasonic propagation fluid). ④Air-filled balloon. ⑤Valve for adjusting water volume. ⑥Valve for adjusting air volume. ⑦Casing. ⑧Detachable ultrasonic probe (7.5–10 MHz). (**B**) Noninvasive system for measuring venous and arterial pressure. The incorporated pressure manometer was an AS ONE M-382 manometer. (**C**) Cross-sectional ultrasonic images: brachial artery before (dotted arrow) and after compression (solid arrow).

Our system uses the same principle as that of Thalhammer et al.^1)^; it is a hybridization of an echo probe, which images the target vein, and a manometer, which obtains pressure values. A fluid-filled diaphragm is placed between the target vein and echo probe that is filled with ultrasonic propagation fluid, and the fluid pressure in the diaphragm is shown on a pressure meter. The fluid is compressed by pressing the probe on the skin while monitoring the ultrasonic images of the target vein until complete compression is confirmed and the corresponding pressure is recorded. Our system presents three key differences compared to the previous system (**Fig. 1A**). First, we used a commercially available and detachable probe, thus minimizing the cost of manufacturing our system. Second, we modified the part that comes in contact with the patient’s skin by using an elliptical diaphragm window that can be aligned with the long axis of the target limb and projecting diaphragm, which helps ensure that the casing of the pressure manometer does not contact the limb. Third, to increase the pressure of the ultrasonic propagation fluid by more than 200 mmHg, we included a pressurizing button in the unit. This feature facilitates the measurement of both venous and arterial pressures. The actual system is shown in **Fig. 1B**. Next, in our system, the fluid in the diaphragm is compressed by pressing the pressurizing button while monitoring the ultrasonic images of the target vein/artery until complete compression is confirmed (**Fig. 1C**). Systolic arterial pressure was defined as the lowest pressure at which the artery remained completely compressed during both systole and diastole. Venous pressure was defined as the lowest pressure that enabled complete compression of the target vein. This approach relies on the fact that the fluid-filled diaphragm, skin, and any subcutaneous fat are sufficiently flexible to ensure that the pressures are equivalent in the diaphragm and target vein/artery. Therefore, target vessels that are close to the surface of the skin are most likely to provide accurate pressure values, and the greater force that is required to compress relatively deep target vessels may result in an overestimated pressure value. For this reason, we examined target veins that were <10 mm from the surface of the skin.

### Prototype components

The incorporated pressure manometer was an AS ONE M-382 manometer (AS ONE Corp., Osaka, Japan: made in 2015). Because its sensor cannot directly measure hydraulic pressure, we inserted a smaller air-filled balloon into the water-filled diaphragm, which then converted the hydraulic pressure into air pressure that could be measured by the manometer. The ultrasonic device was an Aplio 500 duplex device (Toshiba, Tokyo, Japan) with a 7.5-MHz linear array transducer (PLT-704AT; Toshiba).

### Prototype assembly and use

The first step in the assembly of the system is to couple the linear array transducer with the other part of our system. Next, the three-way stopcock connected to the air balloon was connected to allow the pressure manometer and inner air-filled balloon to vent to the atmosphere. Next, the balloon was completely deflated once, and 2 ml of air was injected using a syringe through the stopcock. These steps were required to ensure that the air-filled balloon was fully filled with air at very low pressure. Then, the stopcock was closed so that an appropriate volume of air was retained within the inner balloon. After the air volume adjustment, the level of diaphragm protrusion from the elliptical echo window was adjusted with water so that the diaphragm protruded by approximately 5 mm from the opening of the casing. Additionally, we designed our system so that when the protraction of the balloon was 5 mm, the surface of the diaphragm was very soft, and if the surface of the diaphragm were hard, target veins with low pressure would collapse before we could check the inner surface of the venous lumens with an echo transducer. This calibration process requires approximately 2 min. Assuming that no air or water leakage occurs, recalibration is needed once a week.

For routine medical measurements, the procedure is simple. First, echo gel was applied to the probe, which was then coupled to the system. Then, the probe was positioned perpendicular to and near, but not touching, the skin at the target vessel. In that position, the manometer pressure was set to zero. This zero setting requires approximately 3 sec. Afterwards, the probe was pressed against the skin. If this approach was insufficient to completely compress the target vessel, then the hydraulic pressure inside the diaphragm was increased by pressing the pressurizing button until complete compression was achieved. Our system can achieve venous compression within 3 to 5 sec and arterial compression to measure the systolic blood pressure within 10 to 15 sec. When complete compression was confirmed, the manometer pressure measurement was recorded.

Before clinical testing, to validate the accuracy of our device, a hydrostatic pressure generator was developed to verify whether our system accurately measured venous and arterial pressure. This device consisted of a 2.5 m translucent pipe and ended in a thin rubber balloon (diameter, 6 mm; length, 15 cm). The balloon and translucent pipe were connected by a three-way stopcock, which was also connected to a water-filled injector. The device was placed such that the pipe was perpendicular to the ground, and the balloon was resting on a towel. Then, the balloon was filled with water, and the physician controlled the water level in the pipe by using the stopcock to retain or release water in the injector and modulated the internal pressure of the balloon so that it remained between 0 and 180 mmHg (0 and 243 cm H_2_O).

### Prototype validation

Our first validation step was administration of an in vitro study to determine whether the pressure values from the pressure generator could be accurately measured using the prototype system. Next, we created three scenarios to validate the measurements used in real clinical situations. Each scenario involved measuring pressures in a clinically relevant superficial vein or artery, and these measured values were subsequently compared to values that were measured using conventional techniques. Finally, the correlations between the two sets of pressure values were evaluated using GraphPad Prism 7 software (GraphPad Software Inc., San Diego, CA, USA).

The first scenario involved measuring the CVP 46 times in 14 patients in our intensive care unit who presented with a CVC. Six out of 14 patients were male. The mean age was 76.6±9.8 years (62–92 years). Patient backgrounds included postcolectomy (n=6), postabdominal aortic replacement (n=3), acute abdomen after surgery (n=2), DeBakey IIIb thrombosed acute aortic dissection (n=1), hepatic encephalopathy (n=1), and end-stage carcinoma (n=1). The patients were evaluated in the supine position, and the CVC-associated pressure sensor was disconnected to ensure that the investigator was blinded to the directly measured CVP. In this scenario, the investigator measured external jugular venous pressure (EJVP) as a substitute for CVP by compressing the external jugular vein opposite to the CVC because the distance from this region to the superior vena cava was shorter than that to the veins of the upper limbs, and EJVP was used as the CVP measurement by Thalhammer et al.^1)^ After this measurement was completed, another investigator directly measured the CVP by reconnecting the CVC-associated pressure sensor. When multiple measurements were performed for the same patient, a break of ≥4 h was included.

The second scenario involved measuring the peripheral superficial venous pressure (PSVP) 58 times at the 10-point puncture site in three healthy volunteers (male/female=1/2) with a mean age of 43.6±8.5 years (34–50 years). This parameter might be used to assess venous thrombosis or venous function in the lower limbs. Next, plastic 24-G cannulas were used to secure the venous catheters to the upper limbs (inserted in the cephalic vein near the wrist) and the lower limbs (inserted in the large saphenous vein just above the ankle). Then, subjects were evaluated in various postures with elevated and resting upper limbs, as well as in seated, standing, and dorsal positions of lower limbs. One investigator used the system to evaluate the target veins at 1 cm proximal to the plastic cannula tip. Next, another investigator measured the venous pressure by checking the pressure in the intravenous saline line, which was secured using the plastic cannula. That pressure was subsequently corrected and converted to mmHg.

The third scenario involved measuring the peripheral arterial pressure 28 times in 14 volunteers. Eight out of 14 volunteers were male. The mean age was 56.6±20.7 years (35–97 years). This parameter could be used as an apparatus for measuring blood pressure in daily medical practice or in emergency medical care. The subjects rested for 3 min in the supine position, and then, the investigator used the system to evaluate the brachial artery at the elbow joint. After this measurement, the subject stood and then rested on a chair for 3 min before the systolic blood pressure was evaluated using an automated sphygmomanometer (HBP-9020; Omron Dalian Co. Ltd., Kyoto, Japan). A break of ≥10 min was included when multiple measurements were performed for the same subject.

## Results

The data of the in vitro study confirm the relationship between the pressure values that were obtained using the hydrostatic pressure generator and our system and were analyzed using statistical analysis. In the pressure range of 0 to 180 mmHg, the regression analysis revealed a significant regression equation (y=0.99x+3.13; p<0.001) and an extremely strong correlation (r^2^=1.00) between pressure values. The Bland–Altman analysis between the mean value obtained by our system and the hydrostatic pressure generator indicated a bias of 2.17 mmHg, with a standard deviation of bias of 3.86 mmHg and 95% limits of agreement of −5.40 to +9.74 mmHg.

### Scenario 1 results

**Figure 2:**
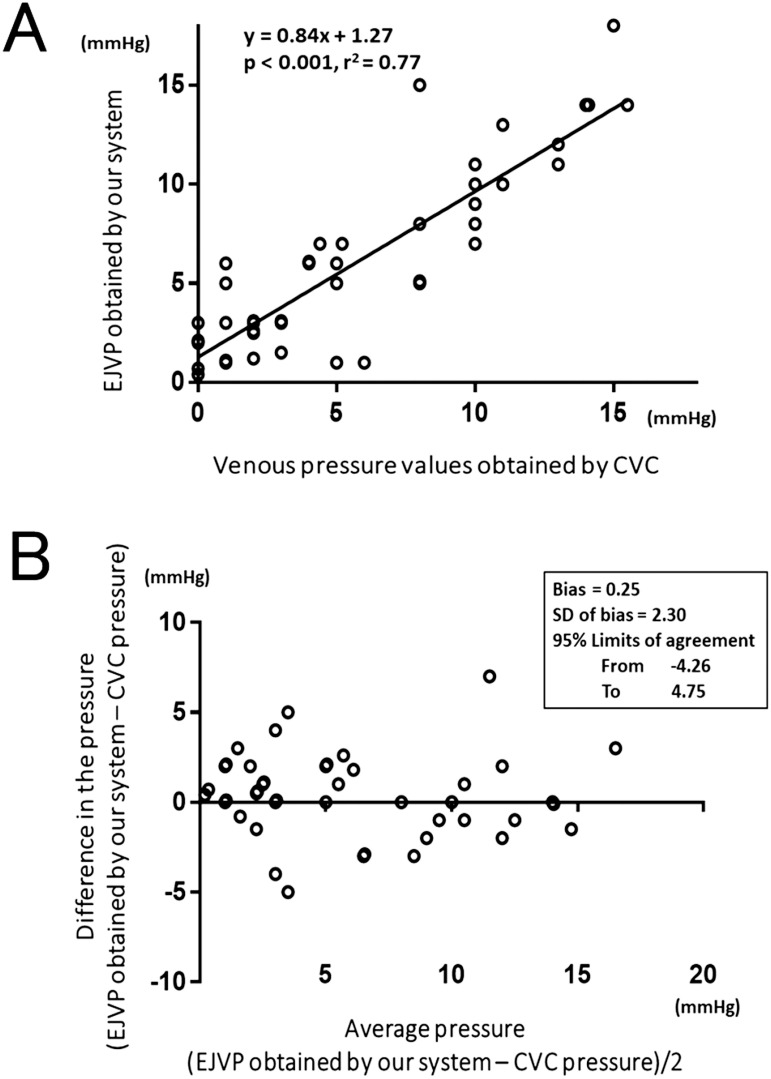
Fig. 2 (**A**) Linear regression: positive correlation between the external jugular venous pressure (EJVP) obtained by our system and the venous pressure value obtained by a central venous catheter (CVC). (**B**) Bland–Altman plot: difference between the EJVP obtained by our system and the pressure value obtained by using a CVC against their average.

**Figure 2A** shows the relationship between the pressure of the superior vena cava (measured using the CVC) and the values of the EJVP measured on the opposite side of the CVC using our system. Regression analysis revealed a significant regression equation (y=0.84x+1.27; p<0.001) and a strong correlation (r^2^=0.77). **Figure 2B** shows the Bland–Altman analysis. The plot shows the differences between the pressure value obtained by our system and the CVP against their average. The bias between the measured values and the average value was only 0.25 mmHg. Based on the results, the standard deviation of bias is 2.30 mmHg, and the 95% limit of agreement is from −4.26 to +4.75 mmHg.

### Scenario 2 results

**Figure 3:**
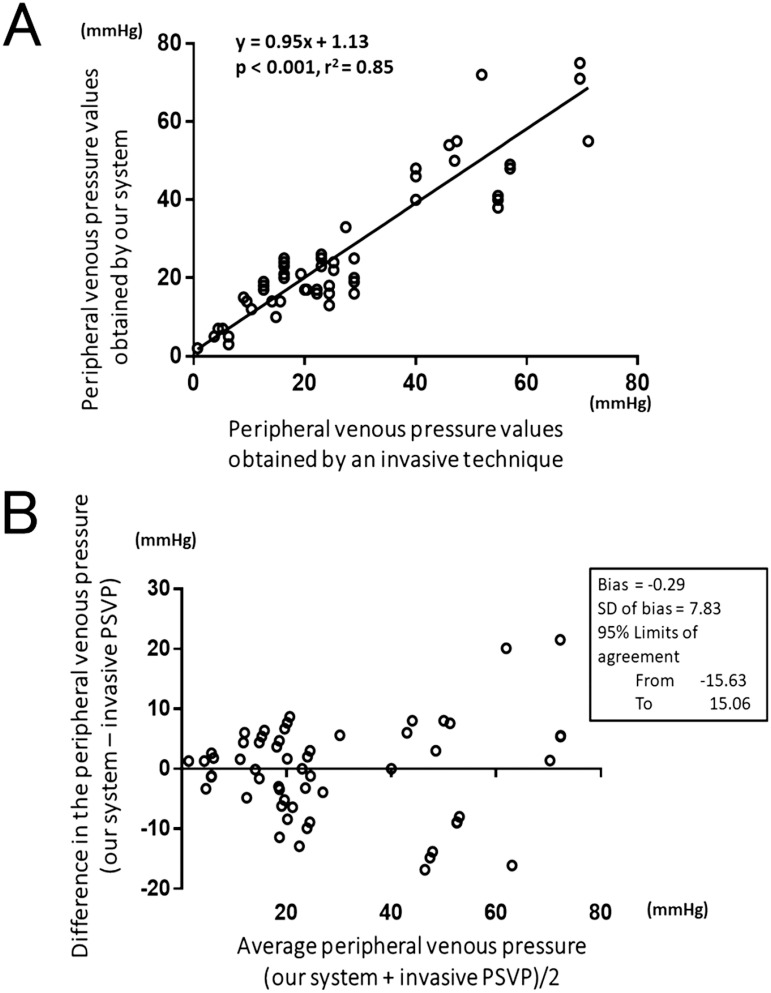
Fig. 3 (**A**) Linear regression: positive correlation between peripheral venous pressure values obtained using an invasive technique and our system. (**B**) Bland–Altman plot: difference between the peripheral venous pressure value obtained by our system and the pressure value obtained by an invasive technique against their average.

**Figure 3A** shows the relationship between the PSVP values that were obtained using the invasive method and our system. Regression analysis revealed a significant regression equation (y=0.95x+1.13; p<0.001) and a strong correlation (r^2^=0.85). **Figure 3B** shows the Bland–Altman analysis. The plot shows the differences between the PSVP obtained by our system and that obtained by the invasive method against their average. Next, the bias between the measured values and the average value was only −0.29 mmHg. Based on the results, the standard deviation of bias is 7.83 mmHg, and the 95% limit of agreement is from −15.63 to +15.06 mmHg.

### Scenario 3 results

**Figure 4:**
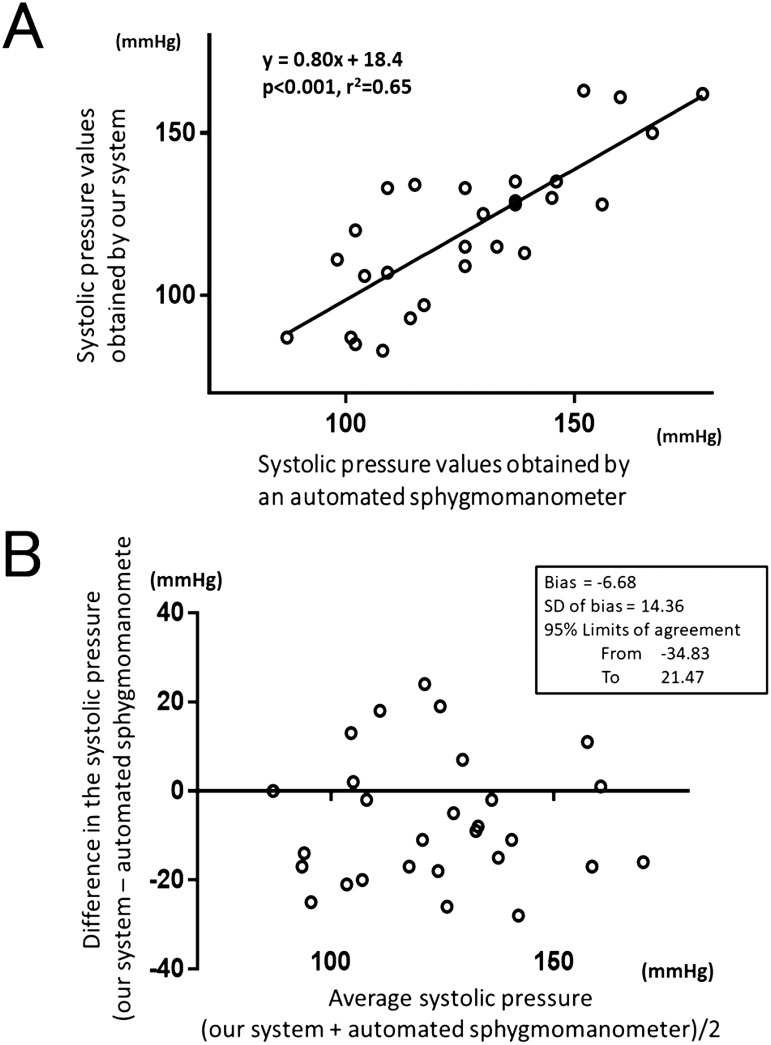
Fig. 4 (**A**) Linear regression: positive correlation between systolic pressure values obtained by an automated sphygmomanometer and our system. (**B**) Bland–Altman plot: difference between the systolic pressure value obtained by our system and the pressure value obtained by an automated sphygmomanometer against their average.

**Figure 4A** shows the relationship between the upper limb systolic blood pressure values that were obtained using an automated sphygmomanometer and the pressure value obtained by our system. Regression analysis revealed a significant regression equation (y=0.80x+18.4; p<0.001) and a good correlation (r^2^=0.65). **Figure 4B** shows the Bland–Altman analysis, and the plot shows the differences between the systolic arterial pressure value obtained by our system and the value obtained by an automated sphygmomanometer against their average. The bias between the measured values and the average value was −6.68 mmHg. Based on the results, the standard deviation of bias is 14.36 mmHg, and the 95% limit of agreement is from −34.83 to +21.47 mmHg.

## Discussion

Several attempts were made to noninvasively measure CVP. One of the methods developed by Xing et al.^2)^ showed a strong correlation between the noninvasive measurements of pressure using ultrasound imaging and the values measured with a central venous catheter, although this technique requires a slightly complex procedure. Next, one of the other methods is based on near-infrared spectroscopy and requires a few minutes of setup.^5)^ Thalhammer et al.^1)^ developed a straightforward method that could be used to rapidly measure central or peripheral venous pressure; however, the Bland–Altman analysis of their results revealed overestimations with their device when high pressure values were measured, especially those over 50 mmHg. Although their measuring technique might be useful for patients without a CVP line and require CVP measurements, its limited range of measurement and high production costs might limit its widespread use. If their system could evaluate all veins and arteries and if its components could be created at a lower cost, the scope of its application would increase. Therefore, we aimed to develop a system that could address these issues. Our design features a detachable ultrasonic probe that is compatible with a commercial pressure manometer, which reduces the cost of the system. Also, we improved the component that contacts the skin of the patient and included a pressurizing button in the unit that can increase the internal pressure of the manometer to more than 200 mmHg. These changes theoretically permit arterial pressures higher than 200 mmHg to be measured.

In our study, the results of the in vitro study using a pressure generator indicated a high coefficient of determination (r^2^=1.0), small bias and small standard deviation of bias, suggesting that this pressure generator is fairly accurate for measuring the vessel pressure near the surface of the skin, even with high pressures of up to 180 mmHg. When our device was used to measure the central (EJVP) and peripheral venous pressures, the results of the Bland–Altman analyses indicated small biases of less than 1 mmHg. Regarding the CVP measurement, the standard deviation of bias was only 2.3 mmHg, and the rate of error measurement exceeding 6 mmHg was only 2.1%. Therefore, although our device might cause a minor error in measurement, a possibility exists that our system is useful for patients who require emergency or repeated measurements and do not present with a CVP line. Regarding the peripheral venous pressure measurements, the coefficient of determination of the correlation between the pressures measured by our system and the pressures measured with the invasive method was 0.85, and the slope of the regression analysis was 0.95. The result indicates that the measurement results of our device were close to full correlation. However, the standard deviation of bias and 95% limits of agreement were not small. One of the reasons for the deviation might be related to the characteristics of our device. As described in the paper by Watson and Wilkinson,^6)^ the CVP itself fluctuates with the cycles of breathing. During the cycle, the CVP increases during expiration and decreases during inspiration. Therefore, when the CVP was recorded in the expiration period, the values indicated a pressure higher than the mean pressure of the breathing cycle, and the pressure was lower in the inspiration period. Because our device records the pressure over only approximately 0.5 sec, the measurement recorded by our device might indicate a higher pressure during expiration and a lower pressure during inspiration periods than the average pressure of the CVC that is generally measured by a pressure sensor, and it reflects the average value across several respiratory cycles. This phenomenon might be avoided by changing the measurement period of our device. Next, one of the other reasons for the relatively large deviation in the PSVP measurement might be the reduction in the venous pressure caused by the venous pump function of the lower extremities in the recording period in both the invasive method and our noninvasive method. Regarding venous pump function in the lower extremities, we can differentiate venous pump function and the type of venous insufficiency using our system by determining the reduction ratio and the duration of reduction of the dorsal venous pressure before and after a tiptoe exercise by using the results of a previous study published by Schanzer and Peirce.^7)^ When the pressure is measured, subtle muscle movements in the lower legs, even without ankle movements, often greatly affect the venous pressure; the magnitude of reduction was more than 10 mmHg, and this effect lasted for approximately 10 to 20 sec. This phenomenon can occur with both the invasive method and our noninvasive method, and the increases and decreases in pressure by 10 mmHg shown in the results of our regression analysis might have been caused by subtle muscle movements that occurred during the measurement with either method. Although some measurement errors may occur, we are using our device to predict the postoperative persistence of leg edema after endovenous laser ablation in patients with varicose veins.

Regarding the arterial pressure measurements, we compared the systolic pressures measured by our device with those measured with the automated sphygmomanometer. The reason that we compared our data with those obtained by a sphygmomanometer was that we intended to compare our device with the most commonly used method worldwide. Although the results indicated a statistically significant correlation, our measurement device indicated a relatively large bias, large standard deviation of bias, and relatively wide 95% limits of agreement, and it underestimated the pressure with respect to that measured by the automated sphygmomanometer. Therefore, this means that our device led to a relatively large error when the value measured by the automated sphygmomanometer indicates was accurate. Various descriptions exist regarding the accuracy of automated sphygmomanometers. First, the manuals of automatic oscillometric sphygmomanometers generally mention that the measurement error of the device is a few percent. Although some clinicians may believe that the error is for an individual blood pressure reading, the errors in the manual do not refer to errors of individual measurement values but the error of an average of individual measurements from 3 blood pressure readings of 85 samples (255 total measurements) against the average value of mercury or that from an aneroid sphygmomanometer read by 2 trained observers.^8)^ A universal standard for the validation of blood pressure measuring devices mentions that currently available automated sphygmomanometers demonstrate a moderate accuracy level and therefore need relatively large sample size, and the device is considered acceptable if the estimated probability of a tolerable error (10 mmHg or less) is at least 85%.^8)^ Because the tolerable error is an absolute value, the measuring device allows for a fairly large measuring width. A national health and nutrition examination survey involving 6.460 cases showed that automated sphygmomanometers indicated an absolute error of 6 mmHg or more and 10 mmHg or more in 38% and 11% of systolic readings and 43% and 16% of diastolic readings, respectively, compared to mercury sphygmomanometers.^9)^ This means that the value measured by an automated sphygmomanometer demonstrate a 27% chance of not being included in the 20 mmHg measurement width. Therefore, an individual reading of an automated sphygmomanometer does not necessarily indicate an accurate measurement value. Thus, even in the case that our device indicated a value close to that measured by a mercury sphygmomanometer, a possibility exists that the systolic values measured by our device exhibit a relatively large error relative to the reading error of automated sphygmomanometers.

Additionally, in recent systemic reviews and meta-analyses conducted by Picone et al.,^10)^ researchers reported that only 50% to 53% of brachial cuff blood pressure (BP) measurements were concordant with intra-arterial brachial BP measurements among patients with BP classified as either prehypertension (systolic blood pressure (SBP) 120–139 mmHg and/or diastolic blood pressure (DBP) 80–89 mmHg) or stage 1 hypertension (SBP 140–159 mmHg and/or DBP 90–99 mmHg). In the report, the overestimation of BP was the predominant issue for brachial cuff comparisons with intra-arterial brachial BP in the pressure range. In our study, the systolic pressure measured with our device was underestimated compared to that measured with the automated sphygmomanometer. This fact might indicate that a chance exists that our device might indicate relatively accurate values when measuring the inner arterial pressure. Regarding arterial pressure, further studies are needed to determine whether our system is clinically useful.

Several potential limitations exist to our study and the system. First, the number of cases included is very small because our device is handmade, and we had only one system that could be used in the study. If we can make copies of our device, conducting multi-institutional joint research might be easy. Second, our device cannot continuously measure the venous and arterial pressure. Third, the size of the device could be reduced to enhance its portability and ease of use. Fourth, object recognition could be incorporated to automatically detect changes in vessel shape, and this adaptation would allow the system to automatically measure central or peripheral venous pressure within approximately 3 to 5 sec and systolic and diastolic blood pressure within 10 to 15 sec. These improvements might also allow our system to be used to noninvasively manage the entire circulatory system.

## Conclusion

Our novel system can noninvasively measure central venous, peripheral venous, and arterial pressures. Next, our system is very easy to use and provides faster measurements than conventional techniques after the system is prepared for measurements. Concerning venous pressure measurements, our device can noninvasively measure not only CVP but also high venous pressures that are higher than 50 mmHg, which are difficult to measure with conventional methods. Finally, regarding arterial pressure, our system can theoretically measure systolic pressure, although improvements to the measurement of diastolic pressure is needed, and further studies are needed to confirm the clinical efficacy of this device.
